# Possible regulation of *Arabidopsis* MYB93 by both SCARECROW and MPK3.

**DOI:** 10.17912/micropub.biology.001986

**Published:** 2026-02-04

**Authors:** Xulyu Cao, Clare Clayton, Bethany Hutton, Nancy McMulkin, Juliet C. Coates

**Affiliations:** 1 College of Resources and Environment, Southwest University Chongqing, China; 2 School of Biosciences, University of Birmingham, Birmingham, B15 2TT, UK

## Abstract

The promoter of the *Arabidopsis* *MYB93* ( *MYB93* ) transcription factor was previously identified in a large-scale screen using the SCARECROW ( SCR ) transcription factor. Independent high-throughput studies also identified MYB93 as a protein-interaction partner of the MAP kinase 3 ( MPK3 ). Here, we validate and extend those observations using RT-PCR, yeast two-hybrid assays and phenotypic analysis. *MYB93* transcript levels were elevated in the *scr-3* mutant and reduced upon expression of SCR-GFP, indicating regulation by SCR. In yeast, the N-terminal domain of MYB93 , but not the C-terminal region, interacted with MPK3 . We also observed that *mpk3-1* mutants exhibited a lateral root phenotype similar to *myb93-1* . Together, these findings support a model in which both *MYB93* gene expression and MYB93 protein function are modulated by SCR and MPK3 , respectively.

**Figure 1. Potential regulation of MYB93 by SCR and MPK3:**
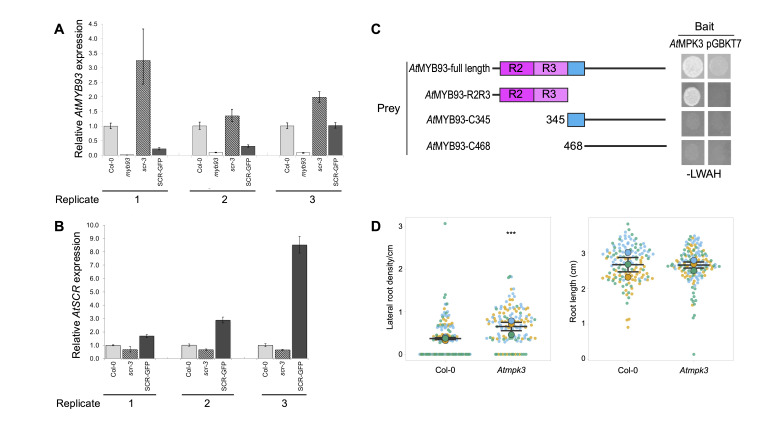
A) Relative levels of *MYB93* ( *AtMYB93* ) mRNA in 7-day old seedlings of wild type (Col-0), *myb93-1* mutant ( *myb93* ), *scr-3* mutant ( *scr-3* ) and *p SCR ::GFP- SCR* in *scr-3* (SCR-GFP) assayed by qRT-PCR.&nbsp; B) Control qRT-PCR experiment showing relative levels of *SCR* mRNA in 7-day old seedlings of wild type (Col-0), *scr-3* mutant ( *scr-3* ) and *p SCR ::GFP- SCR* in *scr-3* (SCR-GFP; (Goh et al., 2016)). In both A) and B) 3 biological repeats are shown: error bars show the upper and lower ranges of fold-change calculated by incorporating the standard deviation of ΔΔCt into the fold-change. Across the 3 biological repeats combined, significant differences were seen with *MYB93* expression between Col-0 and *myb93* (p=0.009) and with *SCR* expression between Col-0 and *p SCR ::GFP- SCR* (p=0.016). &nbsp;The replicates in A) and B) correspond to the same cDNA samples. C) MYB93 ( *At* MYB93 ) interacts with MPK3 ( *At* MPK3 ) in yeast. This interaction is mediated by the N-terminal half of the protein (amino acids 1-345), which includes the R2R3 DNA binding domain (pink boxes) but not the downstream region (blue) unique to the S24 clade ( MYB93 / MYB92 / MYB53 (Gibbs et al., 2014)). pGBKT7, empty pGBKT7 vector control. –LW, yeast grown on medium lacking leucine and threonine to test for plasmid transformation; –LWAH, yeast grown on medium lacking adenine, tryptophan, leucine and threonine to additionally test for protein-protein interaction. D)&nbsp; Lateral root density (left graph) and primary root length (right graph) of wild type (Col-0) and *mpk3-1* ( *Atmpk3* ) mutant 8-day old seedlings. 3 combined biological repeats are shown with data points (small coloured points) for each repeat coloured differently. Larger coloured circles represent the means of the biological repeats and black bars represent the overall mean and standard deviation of the means. A significant difference in lateral root density between Col-0 and *mpk3-1* was seen in a Mann-Whitney test (p = 3.611e ^-10^ ). The number of seedlings per replicate ranges from 38-54.

## Description

The *Arabidopsis* MYB93 ( At1g34670 ) transcription factor is a member of the plant R2R3-MYB transcription factor family (Du et al., 2015) whose gene expression is largely restricted to root endodermal cells overlying developing lateral root primordia and is transiently upregulated during the early stages of lateral root development (Gibbs et al., 2014; Shukla et al., 2021; Voss et al., 2015). MYB93 is a negative regulator of lateral root development as *myb93* mutants show increased lateral root density whilst MYB93-overexpressing plants have fewer lateral roots (Gibbs et al., 2014). MYB93 is part of a small clade of three related proteins, the S24 clade, alongside MYB92 ( At5g10280 ) and MYB53 ( At5g65230 ) (Du et al., 2015; Gibbs et al., 2014).&nbsp; MYB93 , MYB93 and MYB53 , alongside MYB41 ( At4g28110 ), function redundantly to regulate suberin biosynthesis in the root endodermis (Shukla et al., 2021). However, the three S24 genes do not appear to function completely redundantly during lateral root development as *myb93* , but not *myb92* , mutants show elevated lateral root density (Gibbs et al., 2014). In addition, only *MYB93* expression is induced by auxin (Gibbs et al., 2014). Furthermore, via enhanced yeast one hybrid analysis, *MYB93* (but not *MYB92* or *MYB53* ) is implicated in a root signaling network downstream of the endodermal cell identity gene SCARECROW ( *SCR* , At3g54220 ) (Iyer-Pascuzzi et al., 2011; Sparks et al., 2016). Finally, publicly available protein interaction data (Oughtred et al., 2019; Popescu et al., 2009; Wanamaker et al., 2017) suggests that MYB93 has a unique set of interaction partners compared to other S24 clade members. &nbsp;High-throughput protein microarray analysis (Popescu et al., 2009) shows that MYB93 interacts with MITOGEN ACTIVATED PROTEIN KINASE 3, ( MPK3 , At3g45640 ) while other S24 proteins do not. In contrast, MPK6 ( At2g43790 ) interacts with MYB93 , MYB92 and MYB41 (Hoang et al., 2012; Popescu et al., 2009).

&nbsp;

To further explore the upstream regulation of MYB93 , we firstly analyzed the expression of *MYB93* in the *scr-3* mutant using qRT-PCR. We showed that in three separate biological repeats with similar trends, *MYB93* levels are upregulated in *scr-3* mutant seedlings and reduced in a ‘rescue' line ( *p SCR ::GFP- SCR* (Goh et al., 2016)) where *SCR* is re-introduced into a *scr-3* background under the control of its own promoter ( Figure 1A, 1B). This demonstrates that SCR is a likely negative regulator of *MYB93* expression, extending previous protein-DNA interaction studies (Iyer-Pascuzzi et al., 2011; Sparks et al., 2016). Given the highly restricted localization of *MYB93* promoter activity (Gibbs et al., 2014), our data suggest that SCR inhibits *MYB93* promoter activity in most endodermal cells in the root. Interestingly, in a time course transcriptome of root segments induced to form lateral root primordia, *SCR* gene expression is significantly downregulated at 9-12h, immediately before the largest upregulation of *MYB93* at 12-15h (Voss et al., 2015), suggesting that localized *SCR* downregulation may be required for *MYB93* induction.

&nbsp;

To further investigate MPK3 as a potential protein regulator of MYB93 , we tested the interaction of MYB93 with MPK3 in the yeast two-hybrid system. We showed that full-length MYB93 interacts with MPK3 ( Figure 1C ). Furthermore, truncation of MYB93 showed that the N-terminus of the protein (amino acids 2-365, encompassing the R2R3-MYB domain but not the downstream unique motif (Gibbs et al., 2014)) was necessary and sufficient to mediate interaction with MPK3 ( Figure 1C ). The N-terminal region of MYB93 interacted with MPK3 in yeast but the C-terminal region of MYB93 (amino acids 115-365 or 156-365) did not ( Figure 1C ). We next investigated the impact of loss of MPK3 on root development and showed that the *mpk3-1* mutant has elevated lateral root density compared to wild type, similarly to *Atmyb93* ( Figure 1D ; (Gibbs et al., 2014)) but no difference in primary root length ( Figure 1D ). This would position MPK3 as a potential positive regulator of MYB93 during lateral root development, similar to the positive regulation of MYB44 ( At5g67300 ) by MPK3 and MPK6 (Nguyen et al., 2012). A previous study suggested that an *mpk3* mutant does not show a lateral root phenotype (Zhu et al., 2019), although this study was performed on slightly older seedlings (10 days vs 8 days), meaning that differences occurring during early lateral root development may have been overlooked in the 10-day old seedlings. A conditional *mpk3/mpk6* double mutant has fewer emerged lateral roots, suggesting that *MPK3* and *MPK6* could together promote lateral root emergence, via the auxin transporter *LAX3* (Zhu et al., 2019). As *MYB93* inhibits lateral root initiation as well as emergence (Gibbs et al., 2014), this suggests that *MPK3* , on its own or together with *MPK6* , may play contrasting roles at different stages of lateral root development. Collectively, our data suggest that MPK3 may work with MYB93 during lateral root development but not during primary root development.

&nbsp;

In summary, we have extended previous data to show that *MYB93* gene expression is negatively regulated by the endodermal transcription factor SCARECROW . We have also demonstrated that the R2R3-MYB domain of MYB93 interacts with the MAP kinase MPK3 . Furthermore, we have implicated MPK3 in the negative regulation of lateral root development, as a potential positive regulator of MYB93 . Thus, we provide new insights into the mechanism by which endodermal MYB93 regulates lateral root development in a very specific and localized manner.

## Methods

**RNA extraction and cDNA generation**

Up to 100mg plant tissue from pooled 7-day old seedlings was ground in liquid nitrogen using RNAse-free ceramic pestles and mortars. RNA was extracted using an ISOLATE II Plant RNA kit (Bioline, Meridian Biosciences, Memphis, TN, USA). cDNA was generated from RNA using the SuperScript ^TM^ III first-strand synthesis system (Invitrogen, ThermoFisher Scientific, Waltham, MA, USA).

**Quantitative RT-PCR (qRT-PCR).**

qRT-PCR was carried out using Brilliant III ultra-fast SYBR Green low ROX qPCR master mix (600892, Aglient Technologies, Stockport, UK) using a final template concentration of 1ng/µl based on the concentration of RNA added to the cDNA synthesis reaction. The primers used were as follows.

**Table:** 

**Gene**	**5' primer**	**3' primer**
*MYB93*	AAGCTCGCAGATTTGAATAGGTG	ATCTGTACGACCTTGCAAATGC
*SCR*	GCAGATAAGCTTGGCCTGCC	GGAGCTAATCTTTGGAGTAACCAG
*ACTIN2*	TCGTACAACCGGTATTGTGCTG	TAACAATTTCCCGCTCTGCTG
*UBC21*	CGATTCTTGACCAAGATATTCCATC	TTAGAAGATTCCCTGAGTCGCAG

&nbsp;

Primers were used at a final concentration of 200nM-400nM depending on primer efficiency. Reactions were carried out on an AriaMx qPCR machine (Agilent Technologies, Stockport, UK) was used with cycling parameters of 95°C for 10 min, 40 amplification cycles of 95°C for 30 s and 60°C for 1 min. After this, a melt curve cycle (95°C 30 s) was performed, then 65°C for 30 s and ramping back to 95°C for 30 s at a ramp rate of 0.3°C every 2 s to produce a dissociation curve. Three technical repeats per plate were carried out for each sample and three biological replicates were performed overall. Cq values were normalised to *ACTIN2* ( At3g18780 ) and *UBC21* ( At5g25760 ) housekeeping controls and fold changes calculated using the ΔΔCt method (Livak & Schmittgen, 2001). Statistics were performed on ΔCt values of combined replicates: ANOVA followed by a Dunnet's post-hoc test.

&nbsp;

**Yeast two-hybrid assays.**

*MYB93* ( At1g34670 ) full-length and three truncated cDNAs (nucleotides 1-344, 345-1378, 468-1378) were cloned into the pGADT7 vector; full-length *MPK3* (A3g45640) cDNA was cloned into the pGBKT7 vector. The primers used had appropriate restriction sites added for cloning and the primer pairs used were as follows.

**Table:** 

**Gene**	**5' primer**	**3' primer**
*MYB93* full-length	AAAGAATTCGGGAGGTCGCCTTGTTGC	AAAGGATCCCTAAGATATAACGTTCATGAGG
*MYB93* -R2R3	AAAGAATTCGGGAGGTCGCCTTGTTGC	AAAGGATCCTTTCTTCTTTAGATGTGTGTTCC
*MYB93* -C345	AAAGAATTCTTGATCCAGATGGGGATCG	AAAGGATCCCTAAGATATAACGTTCATGAGG
*MYB93* -C468	AAAGAATTCTCCATGCAAGGCGAAGCAG	AAAGGATCCCTAAGATATAACGTTCATGAGG
*MPK3*	AAAGAATTCAACACCGGCGGTGGCC	AAAGGATCCCTAACCGTATGTTGGATTGAGTGC

&nbsp;

2µg of each plasmid (constructs or empty vector controls) was transformed into *S. cerevisiae* strain AH109 Hansen following a small-scale transformation protocol ((Clontech, 2009); Takara Biosciences, Otsu, Japan). Transformed yeast was grown on drop out medium (SD) -LW (DSCK172, Formedium, Swaffham, UK) agar plates for 2-3 days at 30˚C until transformants were observed. Individual transformed colonies were selected inoculated into 50µl of sterile distilled water, 5µl of which was then pipetted onto SD -AHLW (DSCK272 Formedium, Swaffham, UK) agar plates for 2-3 days at 30˚C to test for protein-protein interaction.

&nbsp;

**Plant genotypes, growth conditions and lateral root assays.**

*Arabidopsis* ecotype Col-0 wild type and the *myb93-1* mutant (SALK_131752, NASC ID N631752; (Gibbs et al., 2014)), *mpk3-1* mutant (SALK_151594, NASC ID N869692; (Merkouropoulos et al., 2008)), *scr-3* (Gallagher et al., 2004) and *p SCR ::GFP- SCR* in *scr-3* (Goh et al., 2016) were grown in Levington M3 compost/vermiculite mix at 22˚C under 16h light in a glasshouse. For qPCR, root assays and magenta pot growth, seeds were sterilised for 10 minutes in 10% Parozone ^TM^ Bleach (Jeyes, Hemel Hempstead, UK) followed by 3 rinses in sterile distilled water and resuspension in 200µl distilled water. Seeds were vernalized in the dark at 4˚C for 2 days. For root assays and qRT-PCR, seeds were plated in rows at the top of half-strength Murashige and Skoog (MS) medium (M0404, Sigma-Aldrich, St Louis, Missouri, USA) pH5.6-5.8 with 1% agar. Seedlings were grown vertically for 7 days (qRT-PCR) or 8 days (root assays) and 8-day root plates were photographed. Emerged lateral roots and adventitious roots (roots emerging from the collet) were counted by eye from plates and root length was measured from photographs using the freehand drawing tool in ImageJ (https://imagej.net/ij/). Lateral root density was calculated for each seedling by dividing lateral root number by primary root length. Root data was visualised using SuperPlots ((Lord et al., 2020); https://huygens.science.uva.nl/SuperPlotsOfData/).

Statistical significance for lateral root density and primary root length between Col-0 and *mpk3* was calculated using a pairwise Mann-Whitney U-test.
